# Comparison of Radiomics and conventional SUVr methods for Alzheimer’s disease classification using AV45 PET imaging

**DOI:** 10.3389/fneur.2025.1594470

**Published:** 2025-08-06

**Authors:** Haiyan Gao, Arui Tan, Junhao Wu, Zhen Cao, Ziyang Zhu, Wei Zhang

**Affiliations:** ^1^Department of Nuclear Medicine, Sichuan Provincial People’s Hospital, School of Medicine, University of Electronic Science and Technology of China, Chengdu, China; ^2^Sichuan Provincial Center for Mental Health, Sichuan Provincial People’s Hospital, University of Electronic Science and Technology of China, Chengdu, China; ^3^Department of Nuclear Medicine and PET Center, Huashan Hospital, Fudan University, Shanghai, China; ^4^Siemens Healthineers Ltd., Shanghai, China

**Keywords:** Alzheimer’s disease, AV45, positron emission computed tomography, radiomics, machine learning

## Abstract

**Objective:**

To compare the diagnostic performance of radiomics-based analysis and the conventional standardized uptake value ratio (SUVr) method in classifying Alzheimer’s disease (AD) and non-Alzheimer’s disease (NAD) using AV45 PET imaging.

**Methods:**

This retrospective study included 79 patients diagnosed with AD and 34 patients diagnosed with NAD between July 2023 and August 2024. All patients underwent AV45 PET imaging, and the images were registered to a standard template for the extraction of SUVr metrics, including SUVmaxr, SUVmeanr, and SUVmoder, as well as radiomic features (a total of 660 features) from regions of interest (ROIs) in the brain lobes. Feature importance was ranked using a random forest algorithm, and three models were constructed: an SUVr model, a radiomics model, and a combined model. The classification performance was assessed using receiver operating characteristic (ROC) curve analysis and decision curve analysis (DCA). Model accuracy, sensitivity, specificity, and precision were evaluated using the Mann–Whitney test, DeLong test, and confusion matrices.

**Results:**

There were no significant differences in gender and age between AD and NAD groups (*p* > 0.05). SUVr analysis showed no statistically significant differences in SUVmaxr values in the frontal and occipital lobes between AD and NAD patients, while SUVmeanr and SUVmoder in other lobes exhibited significant differences (*p* < 0.05). The 15 most important radiomic features were primarily concentrated in the temporal, frontal, and parietal lobes, with the highest-ranked features being original_firstorder_Skewness and original_glcm_ClusterShade. The area under the curve (AUC) of the Radiomics model was 0.89 (95% CI: 0.75–0.98), significantly higher than that of the SUVr model (AUC = 0.67, 95% CI: 0.45–0.86, *p* = 0.026). The combined model achieved an AUC of 0.88, showing no significant improvement over the Radiomics model alone. The Radiomics model outperformed the SUVr model in terms of accuracy (88% *vs.* 68%), sensitivity (96% *vs.* 78%), specificity (73% *vs.* 45%), and precision (88% *vs.* 75%). DCA analysis further confirmed the superior diagnostic performance of the Radiomics model.

**Conclusion:**

The radiomics-based approach significantly outperformed the conventional SUVr method, particularly in terms of sensitivity and specificity. This study highlights the potential of radiomics for quantitative PET imaging analysis and its promising clinical applications.

## 1 Introduction

Alzheimer’s disease (AD) is a neurodegenerative disorder characterized by progressive cognitive decline, with pathological hallmarks including amyloid-beta (Aβ) plaque deposition and neurofibrillary tangles formation. The prevalence of AD in individuals over 60 years old is estimated to be 3.7% (1), making it one of the leading causes of cognitive impairment among the elderly, posing a significant burden on individuals, families, and society (2). In the absence of effective prevention and treatment strategies, the number of AD patients is projected to rise dramatically to 13.8 million by 2060 (3). This underscores the increasing need for early identification of high-risk individuals and early diagnosis of AD (4).

Aβ plaque deposition is a hallmark pathological feature of AD and a necessary condition in the AT(N) diagnostic framework. As a non-invasive molecular imaging technique, PET/CT imaging is capable of localizing and quantifying specific biomarkers, thereby providing significant evaluative value for the diagnosis of AD, as well as for assessing disease progression and prognosis (5, 6). However, Aβ PET/CT positivity is not exclusive to AD, as some NAD patients also exhibit similar imaging features, complicating diagnosis and treatment strategies.

Radiomics technology enables the rapid generation of high-throughput imaging features, capturing pathological changes that are imperceptible to the naked eye and may be closely associated with cellular or molecular alterations (5, 7). Currently, radiomics has been applied to AD PET imaging to explore early diagnosis and differential diagnosis through various molecular imaging approaches. Previous studies have demonstrated that Aβ PET radiomic features can serve as novel biomarkers for the clinical application of AD and mild cognitive impairment (MCI) (8). In contrast, the widely used conventional SUVr method is susceptible to multiple factors, including tracer type and analytical processes. Numerous studies have reported that PET radiomics significantly outperforms the SUVr method in diagnostic performance (9–12), suggesting that radiomics may provide a more precise and efficient approach for Aβ PET image analysis, reducing the ambiguities associated with visual interpretation. This study aims to further investigate the potential value of radiomics in the diagnosis and differentiation of AD.

## 2 Materials and methods

### 2.1 Study design

**Figure 1:**
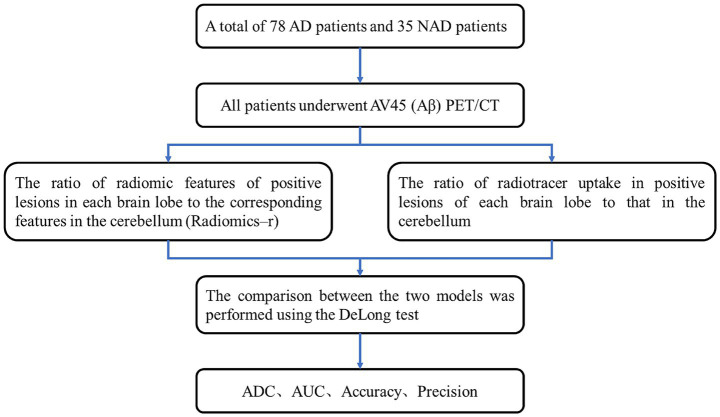
Flowchart of the study.

This retrospective study was approved by the Institutional Review Board, and the study flowchart is illustrated in Figure 1.

### 2.2 Participants

This study was a single-center research conducted by the Sichuan Provincial People’s Hospital. Research data were gathered between July 2023 and August 2024. A total of 113 patients were included, comprising 79 AD and 34 NAD cases. The NAD group includes patients with dementia with Lewy bodies, frontotemporal dementia, progressive supranuclear palsy, vascular dementia, anxiety, and depression. This study received ethical approval (No. 2024.403), with a waiver for written informed consent.

### 2.3 Inclusion criteria

Enrolled subjects with confirmed AD presented clinical manifestations aligning with the 2011 diagnostic guidelines established by the National Institute on Aging and Alzheimer’s Association (NIA-AA) (13, 14). All participants met the diagnostic criteria for probable Alzheimer’s disease.

NAD patients were diagnosed by psychiatrists based on clinical history, physical examination, neuropsychological assessment, neuroimaging, and laboratory tests.

### 2.4 Exclusion criteria

(1) History of stroke with focal neurological deficits. (2) Presence of other neurological disorders that may cause brain dysfunction, including brain tumors, metabolic encephalopathy, encephalitis, multiple sclerosis, epilepsy, and traumatic brain injury. (3) Presence of systemic diseases that may lead to cognitive impairment, such as liver dysfunction, renal dysfunction, thyroid abnormalities, severe anemia, folate or vitamin B12 deficiency, syphilis, HIV infection, and substance or alcohol abuse. (4) resence of intellectual disability or neurodevelopmental disorders.

### 2.5 Imaging acquisition and analysis

Patients were not required to fast before the procedure. A radiotracer dose of 10 mCi per patient was administered via intravenous injection, followed by a 60-min resting period in a quiet, dark, and temperature-controlled environment. Imaging was performed using a Siemens Biograph mCT Flow 64 PET/CT scanner. CT parameters: 120 kV tube voltage, 150 mAs tube current, 2 mm slice thickness, 0.55 mm pitch. PET acquisition: one bed position, 22.1 cm field of view, 15-min acquisition time. Images were reconstructed using the TrueX+TOF method with five iterations and 21 subsets per iteration, yielding a 2 mm slice thickness and 2 mm slice interval.

Image processing was conducted using the Siemens MMWP TrueD nuclear medicine imaging workstation. PET scans were independently reviewed by two experienced nuclear medicine physicians with over 10 years of diagnostic expertise. In cases of disagreement, a senior radiologist provided a final decision. PET/CT positivity was determined based on the Aβ PET diagnostic guidelines (15).

### 2.6 Radiomics and SUVr data acquisition

Currently, PET/CT does not have a standardized T1 template similar to MRI for image matching. According to the Expert Consensus on the Application of Amyloid PET Imaging in the Diagnosis of Alzheimer’s Disease (16), Recommendation 8 suggests that institutions capable of conducting semi-quantitative analysis should use a simultaneously acquired high-resolution 3D-T1WI MRI sequence for subject-specific registration and perform partial volume effect (PVE) correction on the amyloid PET images. In cases where high-resolution MRI is unavailable, preprocessing should be conducted using a tracer-specific standardized brain template (Level II recommendation, Grade B evidence) (16).

In this study, a standardized brain template was used to warp all brain images to a common space, followed by regional brain mapping to extract SUV values and other relevant imaging data for each region of interest.

### 2.7 Registration

Initial registration was performed using Python with the CenteredTransformInitializer method, applying a gradient descent optimizer with the following parameters: SetOptimizerAsGradientDescent (learningRate = 1.0, numberOfIterations = 100, convergenceMinimumValue = 1e-6, convergenceWindowSize = 10). Subsequently, multi-brain registration was conducted using the ANTs (pyants-0.3.0) framework.^1^ PET images were registered to a standardized template, specifically the MNI152_PET_1mm.nii template provided by the BioHistory Group at the University of Copenhagen.^2^

### 2.8 Region of interest selection

After registration, ROI selection was performed, covering the bilateral frontal, temporal, occipital, and parietal lobes. The WFU PickAtlas Tool (version 3.0.5) (17) was used for ROI delineation. Brain region values were extracted using the CL standard mask images (voi_ctx_2mm.nii and voi_WhlCbl_2mm.nii) provided by GAANI (18).

SUVr values were calculated using Python (version 3.7.1) (19). SUVmax: The maximum voxel value within the ROI. SUVmean: The mean voxel value across all voxels within the ROI. SUVmode: The most frequent voxel value within the ROI.

Radiomic feature extraction from the ROI was conducted using Pyradiomics (version 3.0.1). A total of 110 features were extracted per ROI, yielding 660 features in total. The ratio of radiomic features in the positive lesion area to those in the whole cerebellum (WhlCbl) was computed as Radiomics_r.

### 2.9 Feature selection

Patients were randomly divided into a training set (*n* = 79) and a test set (*n* = 34) in a 7:3 ratio. Before feature selection, all features were normalized using Z-score normalization, where each feature value was subtracted by the mean and then divided by the standard deviation (SD). Feature selection was performed based on feature importance ranking using a random forest algorithm.

### 2.10 Radiomics model

The most valuable features were selected from PET images based on random forest feature importance ranking to construct a machine learning model. The selected features were used for random forest model training and validation. Three models were compared in terms of AUC value and classification effectiveness: SUVr alone, Radiomics_r alone, and a combined SUVr + Radiomics_r model.

### 2.11 Statistical analysis

All statistical analyses were conducted using Python (version 3.7.1)^3^ and Pyradiomics (version 3.0.1). Descriptive data were expressed as Mean ± SEM. Differences between groups were assessed using the Mann–Whitney test. The effectiveness of each model was evaluated using ROC curve analysis and AUC values. Model performance comparisons were performed using the DeLong test to assess differences between ROC curves. DCA was used to compare the net benefit of different models.

## 3 Results

### 3.1 Patient characteristics

A total of 113 patients were included in this retrospective study, with 79 patients assigned to the training set and 34 patients assigned to the test set. There were no statistically significant differences in gender or age between the training and test sets (*p* > 0.05; Table 1).

**Table 1:** Patient characteristics.

Features	Training set (*n* = 79)			Test set (*n* = 34)		
	AD (*n* = 55)	NAD (*n* = 24)	*p*-value	AD (*n* = 23)	NAD (*n* = 11)	*p*-value
Female/Male	42/13	14/10	0.107	16/7	6/5	0.398
Age(y)	62.43 ± 9.49	65.08 ± 12.67	0.079	66.17 ± 9.62	68.00 ± 11.24	0.658
Range	46–88	31–81	/	45–81	52–85	/
MMSE	17.56 ± 5.31	18.95 ± 4.04	0.242	16.30 ± 5.92	19.30 ± 4.08	0.482
MoCA	12.85 ± 4.03	14.12 ± 3.97	0.230	12.75 ± 4.71	16.80 ± 3.73	0.017
CDR	0.90 ± 0.54	0.64 ± 0.34	0.110	0.87 ± 0.42	0.80 ± 0.25	0.864

AD, Alzheimer’s disease; NAD, Non-Alzheimer’s disease.

### 3.2 SUV ratios of brain lobes to cerebellum in the training set

A comparison of AD and NAD patients in the training set was conducted using the Mann–Whitney test. The results indicated no statistically significant difference in SUVmaxr values between the two groups in the frontal and occipital lobes. However, significant differences were observed in SUVmaxr, SUVmeanr, and SUVmoder values in the other brain lobes (*p* < 0.05) (Table 2).

**Table 2:** Comparison of SUVr between AD and NAD patients in the training set.

Parameters	Groups	CTX	Frontal	Temporal	Parietal	Occipital
SUVmaxr	AD	1.09 ± 0.16	1.13 ± 0.14	1.05 ± 0.14	1.10 ± 0.16	1.08 ± 0.16
	NAD	0.99 ± 0.11	1.15 ± 0.17	0.99 ± 0.09	1.03 ± 0.09	1.02 ± 0.14
	*p* value	0.006	0.759	0.049	0.027	0.068
SUVmeanr	AD	1.24 ± 0.18	1.10 ± 0.14	1.11 ± 0.13	1.13 ± 0.15	1.17 ± 0.13
	NAD	1.02 ± 0.14	1.01 ± 0.09	1.02 ± 0.08	1.00 ± 0.10	1.05 ± 0.10
	*p* value	0.000	0.002	0.001	0.000	0.000
SUVmoder	AD	1.41 ± 0.29	1.42 ± 0.36	1.34 ± 0.26	1.40 ± 0.32	1.35 ± 0.25
	NAD	1.08 ± 0.21	0.97 ± 0.33	1.04 ± 0.21	1.00 ± 0.26	1.09 ± 0.21
	*p* value	0.000	0.000	0.000	0.000	0.000

### 3.3 Optimal radiomics features

For each patient, 110 radiomic features were extracted from each ROI, including 16 shape features, 19 first-order statistical features, and 75 texture features. The radiomic features used in the model are detailed in Table 3.

**Table 3:** The radiomics features of the PET model.

Model	Filter	Feature class		Number
PET model	Wavelet (HHL)	First Order Features		19
	Wavelet (HHL)	Shape Features		16
	Wavelet (HHL)	Texture Features	GLCM	24
			GLSZM	16
			GLRLM	16
			NGTDM	5
			GLDM	14

### 3.4 Random forest feature importance selection

**Figure 2:**
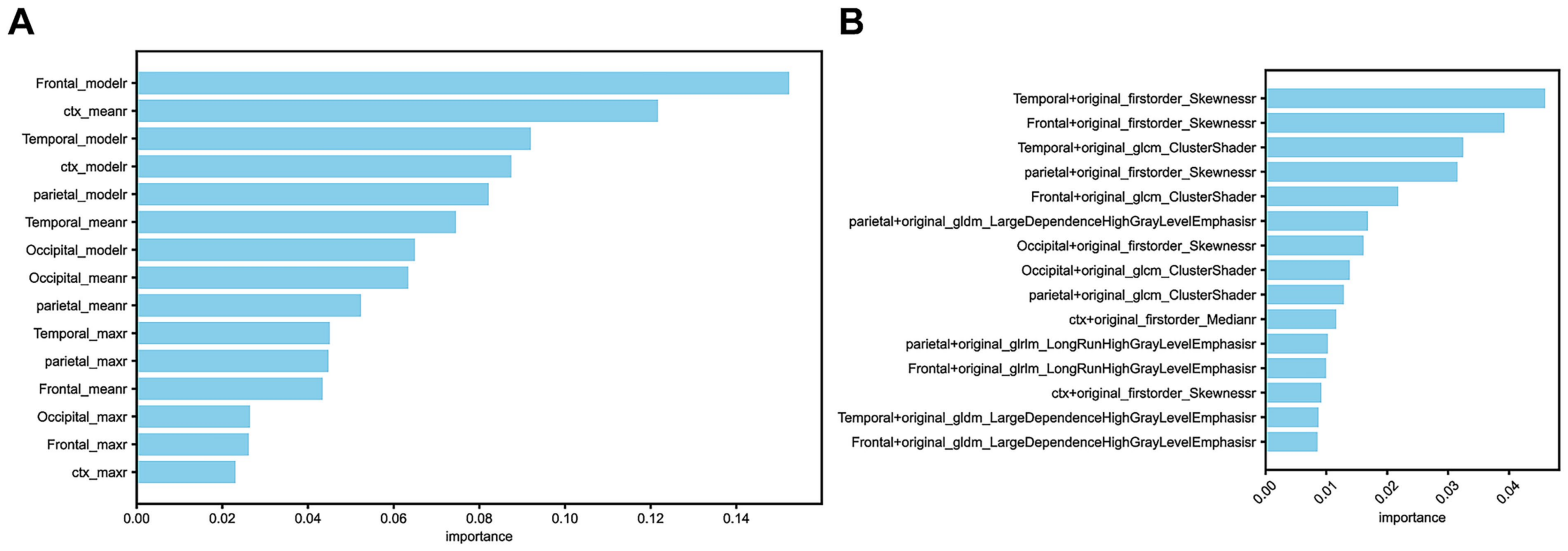
**(A)** The 15 selected features in the SUVr model. **(B)** The 15 selected features in the Radiomics model.

In the SUVr model, a total of 15 feature values (SUVmaxr, SUVmeanr, and SUVmoder) derived from ratios between regions of interest (cerebral cortical and striatal (CTX), bilateral frontal lobes, bilateral temporal lobes, bilateral parietal lobes, and bilateral occipital lobes) and the WhlCbl region were evaluated using a random forest feature importance analysis. Among these, the four most important features were primarily concentrated in the frontal lobe, cerebral cortex and striatum, and temporal lobe, specifically the SUVmeanr and SUVmoder values. In contrast, features from the parietal and occipital lobes, as well as SUVmaxr values, demonstrated lower importance (Figure 2A).

In the Radiomics model, the 15 selected feature ratios exhibited a different distribution of importance. The four most important features were primarily located in the temporal, frontal, and parietal lobes, with the most significant radiomic features being original_firstorder_Skewness and original_glcm_ClusterShade (Figure 2B).

### 3.5 Distribution differences of optimal features in SUVr and Radiomics_r models

**Figure 3:**
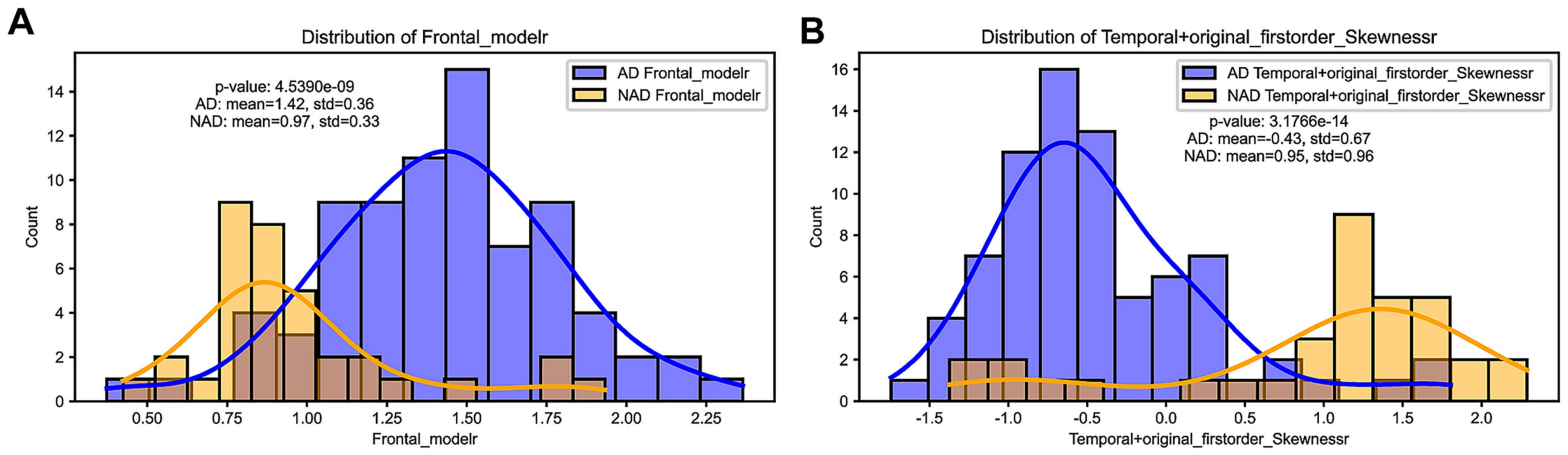
**(A)** Distribution difference of Frontal_moder, the optimal feature selected in the SUVr model. **(B)** Distribution difference of Temporal + original_firstorder_Skewness, the optimal feature selected in the Radiomics model.

The distribution of feature values between the two models showed statistically significant differences (*p* < 0.001). In the SUVr model, the Frontal_moder feature values for AD and NAD patients were 1.42 ± 0.36 and 0.97 ± 0.33, respectively (Figure 3A). In the Radiomics model, the Temporal + original_firstorder_Skewness feature values for AD and NAD patients were 0.43 ± 0.67 and 0.95 ± 0.96, respectively (Figure 3B).

### 3.6 Confusion matrix of SUVr and Radiomics_r models

**Figure 4:**
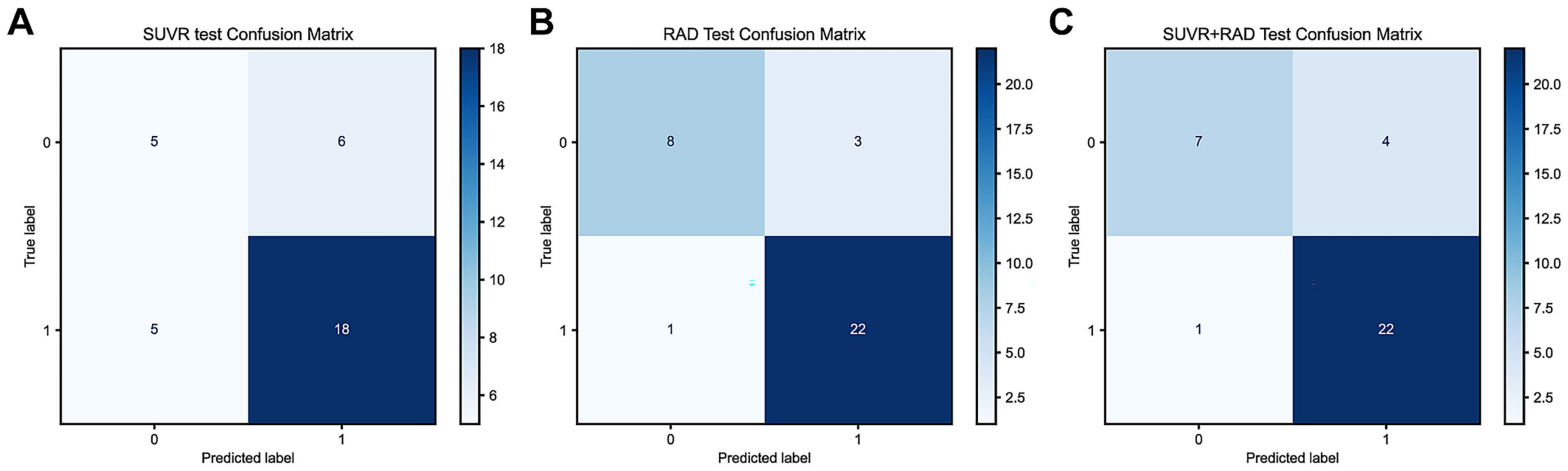
**(A)** Confusion matrix of the SUVr model in the testing set; **(B)** Confusion matrix of the Radiomics model in the testing set; **(C)** Confusion matrix of the Radiomics + SUVr model in the trainning set.

The Accuracy, Precision, Recall, and F1 Score for the SUVr, Radiomics, and Radiomics + SUVr models are as follows: 0.68, 0.88, 0.85, and 0.75, 0.88, 0.85, and 0.78, 0.96, 0.96, and 0.77, 0.92, 0.90, respectively, (Figures 4A–C).

### 3.7 Comparison of ROC and DCA for SUVr and Radiomics_r models

**Figure 5:**
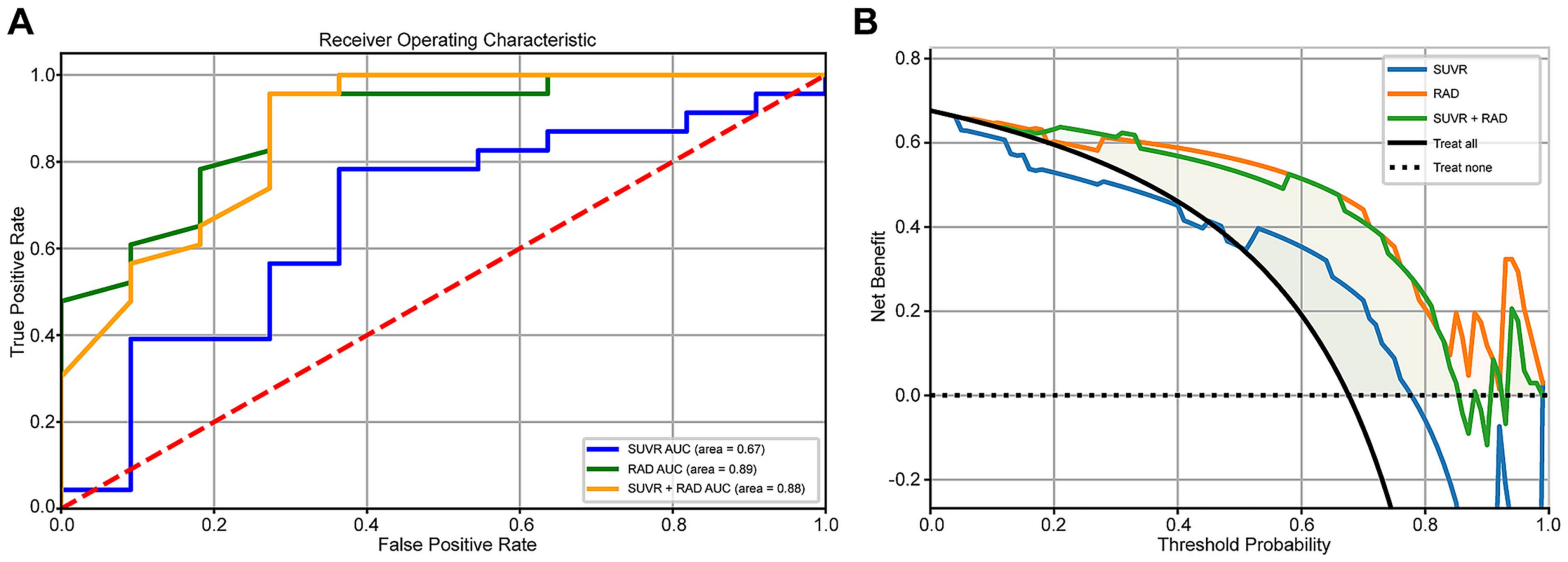
**(A)** Comparison of ROC curves for SUVr, Radiomics_r, and SUVr + Radiomics_r models. **(B)** Comparison of DCA curves for SUVr, Radiomics_r, and SUVr + Radiomics_r models.

For model evaluation, the SUVr model was built using the top four most correlated features, while the Radiomics model incorporated 15 highly correlated features. Additionally, a combined model integrating both approaches was analyzed using ROC and DCA curves. The results demonstrated that the Radiomics_r model achieved the highest AUC value of 0.89 (95% CI: 0.75–0.98), whereas the SUVr and SUVr+ Radiomics_r models were 0.67 and 0.88, respectively. The combined model did not show further improvement in diagnostic performance, with an AUC of 0.88 (95% CI: 0.73–0.99) (Figure 5A). The Radiomics_r model significantly improved the characterization of AV45-positive patients compared to the traditional SUVr approach, with a statistically significant difference in AUC values (*p* = 0.026). Furthermore, the Radiomics_r model outperformed the SUVr model in accuracy, sensitivity, specificity, precision, PPV (Positive Predictive Value) and NPV (Negative Predictive Value) (Table 4). Similarly, DCA analysis confirmed that the Radiomics_r model provided a superior net benefit compared to both the SUVr model and the combined SUVr + Radiomics_r model (Figure 5B).

**Table 4:** The diagnostic ability of each model.

Models	AUC	Accuracy	Sensitivity	Specificity	Precision	PPV	NPV
Radiomics	0.89	0.88	0.96	0.73	0.88	0.88	0.89
SUVr	0.67	0.68	0.78	0.45	0.75	0.75	0.50
SUVr+RAD	0.88	0.85	0.96	0.64	0.85	0.85	0.88

PPV, Positive predictive value; NPV, Negative predictive value.

## 4 Discussion

Alzheimer’s disease (AD) is a severe neurodegenerative disorder, and its diagnosis remains a major challenge in the medical field. The application of Aβ (amyloid-beta) PET/CT imaging tracers has brought significant promise for early disease detection. These tracers enable both qualitative and quantitative assessments of AD by capturing the distribution and concentration of Aβ deposition in the brain. However, the clinical heterogeneity and complexity of AD result in overlapping clinical symptoms and imaging features with non-AD patients, which complicates accurate diagnosis, prognosis, and targeted treatment (20, 21). Therefore, developing more advanced biomarkers and models to improve metabolic information and predictive ability are still crucial for distinguishing AD from non-AD (9).

In recent years, the emergence of radiomics has provided an advanced approach to disease diagnosis. By extracting high-throughput imaging features, such as texture, morphology, and grayscale distribution, radiomics captures both low-order and high-order statistical attributes of imaging data, making it particularly suitable for complex, heterogeneous, and multifaceted diseases. This has improved the accuracy of clinical diagnosis, prognosis, and prediction, and has increasingly attracted attention in the study of brain diseases (22, 23). The increasing application of machine learning has further strengthened radiomics analysis, allowing for more comprehensive models to handle high-dimensional and multi-variable data compared to traditional methods. These models facilitate disease prediction and accurate classification of overlapping symptoms, thereby aiding clinical decision-making (24). More recently, deep learning has been increasingly used for neuroimaging tasks involving classification and prediction. Compared to traditional approaches, deep learning models have the advantage of minimal inference time while eliminating the need for complex image preprocessing steps (25). These advancements provide an optimal solution for AD diagnosis and differentiation and have demonstrated promising classification performance (26). The integration of radiomics and machine learning offers a vast research potential for novel imaging data analysis methods.

This study utilized Aβ PET/CT imaging data to extract 15 radiomic features, identifying four features with strong correlations to AD diagnosis, primarily located in the frontal, temporal, and parietal lobes. The most significant features included original_firstorder_Skewness and original_glcm_ClusterShade. original_firstorder_Skewness reflects the skewness of pixel intensity distribution, indicating asymmetrical metabolic or pathological deposition patterns in specific brain regions. Meanwhile, original_glcm_ClusterShade, derived from the gray-level co-occurrence matrix, quantifies texture complexity and captures microstructural variations in brain gray matter. This complements the limitations of the SUVR single quantitative value (10). And has a significant association with the clinical diagnosis than SUVR (27). The distribution of these radiomic features closely aligned with findings from the SUVr method (involving the frontal, temporal, and parietal lobes), which is also consistent with previous studies (8, 28, 29), highlighting the critical role of these brain regions in AD diagnosis. Additionally, in the SUVr method, moder emerged as a particularly strong diagnostic feature. As a quantitative metric, moder reflects the mode signal intensity within a specific brain region, providing a detailed characterization of regional Aβ pathology distribution.

Comparing radiomics and SUVr methods, the Radiomics_r model demonstrated significantly superior diagnostic performance based on ROC curves and DCA decision curve analysis, achieving an AUC of 0.89 and an accuracy of 0.88. These findings align well with literature reports (10, 11, 30, 31), where AUC values for AD differentiation typically approach 0.9, and PET-based classification of AD versus normal controls achieves accuracy rates of 80–90% (32, 33). In contrast, the SUVr model exhibited lower AUC, sensitivity, specificity, and accuracy, with specificity being particularly low (0.45). This is consistent with prior findings, as A*β* PET has been reported to have relatively low specificity (below 60%) (34). Similarly, a meta-analysis by Morris et al. (35) found that while Aβ PET is highly sensitive for detecting AD and demonstrates good overall diagnostic efficacy, its specificity remains moderate. This is largely due to a high false-positive rate, as other types of dementia, such as dementia with Lewy bodies (DLB) and frontotemporal dementia (FTD), may also present AD-like pathological changes. Additionally, SUVr values often fall within a research-defined range, with whole-brain cortical SUVr thresholds typically between 1.2 and 1.5 (36). Chanisa et al. (37) observed that β-amyloid plaques tend to spread from the cerebral cortex to the cingulate and precuneus regions, with the highest 11C-PIB deposition in the anterior and posterior cingulate gyri. Their study also suggested that regional SUVr-based diagnosis provides greater sensitivity and specificity than whole-brain SUVr, with regional SUVr cut-off values between 1.46 and 1.81. This highlights the substantial overlap between different pathological conditions, which our study aims to address by distinguishing Aβ PET-positive lesions in AD and NAD patients. Our results confirm the irreplaceable advantage of radiomics in overcoming this challenge.

Interestingly, the combined SUVr + Radiomics_r model exhibited lower diagnostic performance than the Radiomics_r model alone. This may be attributed to fundamental differences in how SUVr and Radiomics_r features characterize imaging data, suggesting that radiomics not only enhances the differentiation between AD and NAD patients but also provides a more precise capture of key disease characteristics.

It is worth noting that despite the inferior overall performance of the SUVr model compared to radiomics, there remains potential for improving its diagnostic capability. In cases where full radiomics analysis is not feasible, selecting moder values from the frontal, temporal, and parietal lobes as supplementary indicators could enhance the reliability of SUVr-based diagnosis. This finding provides new insights for optimizing traditional methods.

This study has several limitations. First, radiomics analysis relies on large, high-quality datasets, and our study was limited by a relatively small sample size and single model fitting. Larger datasets are needed to validate the generalizability of our findings and assess results stability through cross-validation. Second, we only extracted PET-based features, as CT and PET/CT standardized brain templates were unavailable, preventing the extraction of multimodal features. Furthermore, MRI imaging data were not incorporated, despite its importance in structural brain analysis. The inclusion of MRI data in future studies could provide a more comprehensive assessment of radiomic features. Additionally, variations in tracer affinity, pharmacokinetics, region of interest selection, reference regions, and imaging acquisition parameters (e.g., tracer dosage, scan timing, and image reconstruction techniques) may lead to inconsistencies across studies, emphasizing the need for standardized quantitative methodologies to ensure reproducibility across different research centers.

## 5 Conclusion

This study demonstrates the significant potential of radiomics and machine learning in Aβ PET/CT-based AD diagnosis and differentiation. Radiomic features derived from Aβ PET imaging could serve as novel neuroimaging biomarkers for clinical applications in AD. Additionally, Aβ PET imaging enables continuous monitoring of brain Aβ burden dynamics, establishing correlations with disease progression and facilitating treatment guidance and therapeutic efficacy evaluation.

## Data Availability

The original contributions presented in the study are included in the article/supplementary material, further inquiries can be directed to the corresponding author/s.

## Footnotes

^1^ https://github.com/ANTsX/ANTsPy

^2^ https://researchprofiles.ku.dk/en/publications/a-fdg-pet-template-in-mni-space-aligned-to-the-mni152-atlas-this

^3^ https://www.python.org/
